# The influence of cerebral small vessel diseases on the efficacy of repositioning therapy and prognosis of benign paroxysmal positional vertigo

**DOI:** 10.7150/ijms.73080

**Published:** 2022-07-04

**Authors:** Jian Zang, Xuejun Jiang, Shuai Feng, Hongyang Zhang

**Affiliations:** Department of Otolaryngology, the First Affiliated Hospital of China Medical University, Shenyang 110001, China.

**Keywords:** Benign paroxysmal positional vertigo, BPPV, Cerebral small vessels diseases, CSVD, Recurrence, Residual dizziness, White matter hyperintensity

## Abstract

**Background:** Although vascular risk factors have been found to be closely related to the development of benign paroxysmal positional vertigo (BPPV), the relationship between BPPV and cerebral small vessels diseases (CSVDs) has rarely been discussed in literature. This study set out to investigate the efficacy of repositioning therapy and prognosis among BPPV patients with CSVDs.

**Methods:** We enrolled 553 BPPV patients who had undergone brain MRI, and categorized them into two groups based on the presence or absence of CSVDs. After controlling for other confounders using a propensity score matching (PSM) approach, we compared the incidence of recurrence and residual dizziness (RD). Then, we analyzed the recurrence rate and RD incidence in 176 BPPV patients with CSVDs, and assessed potential risk factors.

**Results:** White matter hyperintensity (WMH, 72.2%) and lacunar infarction (LI, 65.9%) were the two CSVDs that were present in the highest proportion among the BPPV patients. The incidence of RD in patients with CSVDs was significantly higher compared to subjects without CSVDs. Patients with RD (n=100, 56.8%) were older, had more severe WMH, and had a higher incidence of brain atrophy; age and higher Fazekas score were independent risk factors. Among the recurrent patients (n=61, 34.7%), the ages were older, the Fazekas score of WMH was higher, and number of LIs was increased; age was the sole independent risk factor.

**Conclusion:** BPPV patients with a combination of CSVD comorbidities, especially elderly patients with WMHs, are more likely to develop RD, which needs to be paid more attention.

## Introduction

Benign paroxysmal positional vertigo (BPPV), a peripheral vestibular disease, refers to repeated episodes of vertigo that are triggered by a change in head position 1. It is the most common peripheral vertigo disease in clinic, with a lifetime prevalence of 2.4%, a 1-year prevalence of 1.6%, and a 1-year incidence of 0.6% 2. BPPV accounts for 17% to 42% of vertigo patients. The high incidence of BPPV seriously endangers people's health 3. Most BPPV patients do not think that it is a malignant due to its name, but it can have a serious impact on patients' daily activities. BPPV can have a huge impact on patient's quality of life, and is closely related to patients' anxiety and depression, due to an increased risk of falls 2, 4, 5.

In recent years, some studies have shown that vascular risk factors are closely related to the occurrence and prognosis of BPPV 6, 7. The labyrinthine artery of the inner ear is a tiny terminal artery. Small vessel lesions that directly damage the tiny arteries of the inner ear lead to microcirculation disorders, which can cause damage to the blood supply vessels of the inner ear, leading to the shedding of otoliths, which may be involved in BPPV pathogenesis 7-9. Abnormal cerebral small vessels, including arterioles, branch arterioles, capillaries and venules, can directly damage blood supply, leading to microcirculatory disorders 10. However, there have been almost no reports regarding the relationship between BPPV and cerebral small vessels diseases (CSVDs).

Brain MRI is not been routinely conducted for patients with BPPV. However, the pathogenesis of acute vertigo can sometimes be very complicated, especially in middle-aged and elderly patients with more basic diseases and a complicated past medical history. Therefore, sometimes we may need an MRI to check whether such patients have other brain diseases 11, 12. In clinical practice, we find that many BPPV patients have lesions of cerebral small vessels, including white matter lesions. As we know, MRI manifestations of CSVDs include white matter hyperintensity (WMH, T2WI and flair R), lacunar infarction (LI), enlarged perivascular space (EPVS), cerebral microbleed (CMB) and brain atrophy 13. Therefore, we sought to determine the distribution of these CSVDs in BPPV patients. We also set out to investigate the influence of CSVDs on efficacy of repositioning therapy and prognosis of BPPV patients. To our knowledge, there are no studies on this topic yet.

Although great progress has been made in the diagnosis and treatment of BPPV in recent years, there are different prognoses for the same treatment method 14-16. This is particularly true for the recurrence and residual dizziness (RD) of BPPV, which seriously affect quality of life. Therefore, what remains to be asked is whether the presence of CSVDs and what types of CSVDs have an effect on the prognosis of patients with BPPV? This study investigated the relationship between BPPV and CSVDs, and the influence of CSVDs on the efficacy of repositioning of BPPV which can help us find effective treatment and improving prognosis.

## Materials and methods

### Patients

This is a retrospective study that was granted approval by the Ethics Committee of the First Affiliated Hospital of China Medical University, and was granted a waiver of written informed consent based on the study's retrospective design and anonymous data collection. The study was performed in accordance with the declaration of Helsinki.

Patients who were diagnosed with BPPV and treated at the Department of Otorhinolaryngology, the First Affiliated Hospital of China Medical University from December 2016 to May 2020 were enrolled retrospectively. All patients had undergone brain MRI examination for complaining of acute-onset dizziness at emergency department or neurology department before been transferred to the vertigo clinic of ENT department. The inclusion criteria are as follows: 1. recurrent episodes of transient vestibular vertigo induced by gravity-related head position changes; 2. Dix-Hallpike test or Roll test can induce vertigo and characteristic nystagmus; 3. the patients were clearly conscious and able to cooperate in the canalith repositioning procedure (CRP) treatment, with effective results; 4. MRI was performed within 1 week after onset; 5. denied a previous history of dizziness.

The exclusion criteria were as follows: 1. insufficient medical records; 2. patients who failed complete follow-up; 3. history of head trauma or head surgery; 4. intracranial tumor; 5. inner and middle ear diseases including Ménière's disease, vestibular migraine, sudden deafness, vestibular neuritis, otitis media; 6. use of ototoxic drugs; 7. patients with bilateral or multiple semicircular involvements; 8. cerebrovascular diseases due to cardiac and large vessel obstruction; 9. progressive multifocal leukoencephalopathy; 10. history of atrial fibrillation, myocardial infarction, valvular heart disease, infective endocarditis, myocarditis, mitral stenosis, or aneurysm; 11. severe extracranial and intracranial large blood vessels diseases; 12. evidence of known vasculitis; 13. multiple sclerosis, or other metabolic or toxic leukoencephalopathy; 14. osteoporosis.

### Intervention and follow-up

The eligible subjects were separated into two groups based on presence of CSVDs. Age, gender, brain MRI results, features of BPPV (including involved side and canals), duration of BPPV, times of CRP, as well as the recurrence and RD of BPPV after successful treatment were recorded. We used a propensity score analysis to match CSVDs patients with non-CSVDs patients at a ratio of 1:1.

Patients with posterior canal (PSC) BPPV were treated using the Epley maneuver. The Barbecue maneuver was performed for treatment of geotropic horizontal canal (HC) BPPV. The Gufoni maneuver was used for apogeotropic HC BPPV. Details of the Epley, Gufoni, and Barbecue maneuvers were described in clinical practice guidelines of BPPV established by the AAO-HNSF (American Academy of Otolaryngology-Head and Neck Surgery Foundation) 3. In order to treat anterior canal (AC) BPPV, we used Yacovino maneuver 17.

RD was defined as a feeling of unsteadiness and/or light-headedness and/or dizziness in absence of true vertigo and nystagmus, despite successful repositioning. The recurrence was defined as further BPPV episodes similar as the primary attack and the positive result of positional nystagmus test during follow-up. Three days after CRP treatment, BPPV patients returned to clinic for evaluation of efficacy. A successful CRP was defined as an absence of both positional vertigo and positional nystagmus 3. The patients who failed to the repositioning procedure were reevaluated and underwent CRP every 3 days until the positioning nystagmus disappeared. We recorded the number of times CRP was performed. Follow-up was done at one year after treatment. Then, we investigated the recurrence and RD by checking the patient's outpatient records and telephone.

### MRI protocol

A 3-T system scanner (MAGNETOM Verio, SIEMENS, Erlangen, Germany) was utilized for MRI. Parameters of spin-echo T1-weighted imaging (T1WI) included repetition time (TR) of 2000 ms, echo time (TE) of 17 ms, slice thickness of 5 mm, and slice distance of 1.5 mm. Parameters of spin-echo T2-weighted imaging (T2WI) included TR of 3500 ms, TE of 95 ms, slice thickness of 5 mm, and slice distance 1.5 mm. Parameters of fluid attenuated inversion recovery (FLAIR) sequences included TR of 8000 ms, TE of 104 ms, slice thickness of 5 mm, and slice distance of 1.5 mm. Susceptibility weighted imaging(SWI) included TR of 27 ms, TE of 20 ms, slice thickness 5 mm, and slice distance of 1.5 mm. The field of view was 230 mm × 230 mm, while the construction matrix was 192×256, and standard T2 and FLAIR for T2WI and FLAIR.

### Manifestations of CSVDs on MRI

The MRI features of vasogenic WMH are high-intensity signal on T2 weighted (T2WI) images and Flair sequences, which showed punctate, patchy or confluent high intensity signal in dots, patches or fusion, and iso-signal or low signal on T1 weighted (T1WI) images. We further graded WMH according to the Fazekas 18 rating scales criteria. WMH was then divided into either periventricular white matter hyperintense (PWMH) or deep white matter hyperintense (DWMH), with regards to the location. PWMH showed hyperintense signals surrounding the ventricles in T2WI and isointense or hypointense signals in T1WI. PWMH was graded as 0=absence, 1=“caps” or pencil-thin lining (≤5 mm), 2=smooth “halo” (6-10 mm) and 3=large conglomerate lesions (>10 mm). DWMH showed balanced and irregular hyperintense signals in subcortical white matter, as well as beside the watershed area in T2WI and isointense or hypointense signals in T1WI. DWMH was graded as 0=absence, 1=punctate foci, 2=beginning confluence of foci, and 3=large confluent areas, which has affected most white matter areas. The total score of WMH obtained was the sum of both locations scores (0-9 points).

LI is manifested as a well-defined lesion with a diameter of 3-15 mm, T1WI with low intensity signal and high intensity signal on T2WI, and needs to be distinguished from EPVS (the diameter of EPVS is usually less than 3 mm). We determined and counted the total number of LIs in each patient.

The imaging definition of CMB is on a SWI sequence, which shows a uniform, round, well-defined boundary, low-signal focus/lesion with a diameter of 2-5 mm and no peripheral edema. It is necessary to exclude the vascular flow void and calcification without hemorrhage.

The EPVS manifests as a gap consistent with the course of the blood vessel. In the vertical plane of the blood vessel, there is often a round or oval cerebrospinal fluid signal with a diameter of less than 3 mm. The signal intensity is low on T1WI images, high-signal on T2WI and low-signal on Flair images.

Brain atrophy is characterized by a reduction in brain volume, which is reflected by an enlargement of ventricles and sulcus.

All brain MRI were reviewed and evaluated by an experienced radiologist.

### Statistical analyses

Software SPSS 25.0 was utilized for statistical data analyses. We used a propensity score analysis to match CSVDs patients with non-CSVDs patients at a ratio of 1:1 with a match tolerance 0.02 and based on age, gender, laterality, previous BPPV history, duration of BPPV, number of CRPs and involved canal. Categorical variables were represented as either counts or percentages. For comparison among groups, the Chi-square test, Fisher exact test and McNemar test were used. Independent-samples t-test and paired t-test were used for continuous variables which were normal distribution and described by mean ± standard deviation (SD). The Mann-Whitney U and Wilcoxon test were used for continuous variables with an abnormal distribution and described by median (quartile range, QR). A binary logistic regression model helped determine risk factors for recurrence and RD of BPPV. All variables with *P*<0.05 at univariate test statistics were regarded as covariates and included in multifactorial logistic regression model as potential predictors. The odds ratio (OR) and 95% confidence interval (CI) were calculated. A difference was considered statistically significant if *P* <0.05.

## Results

### Basic characteristics of patients

**Figure 1:**
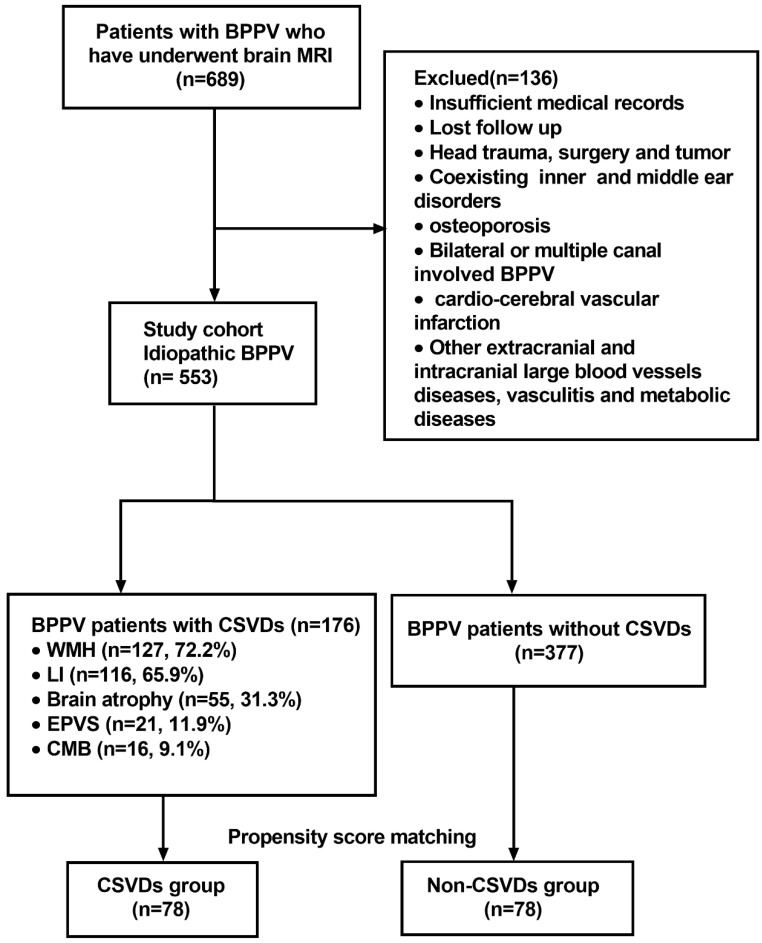
** Flow chart of the study design.** Abbreviations: benign paroxysmal positional vertigo (BPPV); cerebral microbleed (CMB); cerebral small vessel disease (CVSD); enlarged perivascular space (EPVS); lacunar infarction (LI); white matter hyperintensities (WMH).

Overall, 689 patients who met the inclusion criteria were initially enrolled in the study. According to the exclusion criteria, 136 patients were then excluded at screening. Finally, a total of 553 patients were found to be eligible. Among them, 176 BPPV patients whose brain MRI showed the presence of CSVDs, including 74 males and 102 females with an age range of 31-91 years (median: 68). The distribution of CSVDs among 176 BPPV patients with CSVDs is shown in Figure 1. After matching on the propensity score, 78 patients in the CSVDs group and 78 patients in the non-CSVDs group were included in the study. A flow chart describing the study design is depicted in Figure 1.

### Comparison of clinical parameters between BPPV patients with and without CSVDs

After matching the two groups using propensity score analysis based on age, gender, laterality, previous history of BPPV, duration of BPPV, CRP times and involved semicircular canal types, we found a statistically significant difference in RD. Although there were more cases of 1-year recurrence in the CSVDs group, the difference was insignificant (Table 1).

### Analysis of recurrence rate and RD incidence in BPPV patients with CSVDs

As WMH (72.2%) and LI (65.9%) are the two CSVDs with the highest proportion in BPPV patients (Figure 1), we further scored WMH severity and counted the number of LIs in each patient. Among the 176 patients, 61 patients (34.7%) had a recurrence during the 1-year follow-up period. In the recurrent patients, the median age was older, the Fazekas score of WMH was higher, and number of LIs was more (Table 2). There were 100 patients (56.8%) who developed RD after the CRP treatment. The patients with RD had higher age, higher Fazekas score of WMH and higher incidence of brain atrophy (Table 2).

### Multivariate logistic regression analysis of risk factors for recurrence and RD in BPPV patients with CSVDs

In multivariate logistic regression analysis for RD, when other factors remained unchanged, for every one point increase in Fazekas score of WMH, the probability for RD occurrence was 1.078 times higher compared to if the Fazekas score did not increase (OR=1.078, 95%CI: 1.041-1.116; p=0.010). Similarly, for every year increase in age, the probability for RD occurrence was 1.397 higher times compared to if the age did not increase (OR=1.397, 95%CI: 1.197-1.63; p=0.008). Hence, it can be concluded that increased severity of cerebral white matter lesions and age are risk factors for RD. Among the multivariate logistic regression analysis for recurrence, we found the increased age was an independent risk factor (OR=1.105, 95% CI: 1.060-1.152; p=0.012).

## Discussion

CSVDs are common among middle-aged and elderly people, and often are accompanied by no obvious clinical symptoms 19, 20. In this study, we enrolled patients with BPPV who had undergone brain MRI as research subjects. We found that CSVDs was related to RD and recurrence of BPPV among BPPV patients.

The concomitant CSVDs among BPPV patients in this study include WMH, LI, EPVS, CMB and brain atrophy. Among them, WMH was the most common CSVD, which is consistent with the findings of Cha and others. Through investigation of the brain MRI of BPPV patients in the emergency department, Cha *et al.* found that about 70% of patients had WMH 12. There were many factors causing WMH, among which hemodynamic changes, which are recognized by most researchers, leads to insufficient perfusion of deep brain tissue, decrease of blood flow, and finally ischemia and demyelinating changes in an area dominated by arterioles 21, 22. WMH is an indicator of chronic microvascular ischemia 21. Additionally, in this study, LI is also a common manifestation of CSVDs, which mostly located in the basal ganglia, frontal lobe, and parietal lobe. Reuck found that 25% of BPPV patients had small old cerebral infarcts on imaging, while none of them had a prior history of stroke 23. Von Brevern *et al.* also noted that stroke can be an independent risk factor for BPPV 2. WMH and LI are considered to be signs of small vessel disease in the brain, which can be detected by brain MRI 13.

Pathological changes, including cerebral small vessel stenosis or occlusion, are mostly chronic, and there are no obvious stroke events in clinic 10. However, small vessel endothelium has been damaged, which causes a decrease in cerebral blood flow. In addition to causing brain tissue hypoperfusion and CSVDs, it can also cause ischemia of the inner ear 23, 24. The inner ear is an organ with strong aerobic metabolism and is sensitive to ischemia 9, 23. In particular, ischemia can cause local microcirculation disturbances to the vestibule, damage the elliptic sac, make otoliths more likely to fall off, and at the same time, affect the absorption of otoliths in the endolymph, which causes BPPV recurrence and occurrence of RD 7-9.

Frequent recurrence of BPPV seriously affects the quality of life and makes patients anxious. Hence, the early identification and prompt management of BPPV is very important. We excluded patients with a history of head trauma, Meniere's disease, migraine, osteoporosis, simultaneous bilateral or multiple canals involvement and other factors that could lead to the recurrence of BPPV 7, 14, 25-27. In our study of 176 BPPV patients with CSVDs, the recurrent patients had higher age, higher Fazekas score of WMH, and more number of LIs. It has been found that the recurrence of BPPV is closely related to vascular risk factors, such as high hypertension, hyperlipidemia, diabetes, and smoking, among others 14, 27-31. At the same time, they are many risk factors that are related to the onset of CSVDs 20, 32. These vascular risk factors may cause insufficient perfusion of the anterior vestibular artery by changing blood vessels, leading to circulatory abnormalities of inner ear and causing damage to the macula and otoconia detachment 7-9. Therefore, it is not difficult to understand that WMHs and LIs were more severe in the patients with recurrence. However, multivariate logic regression analysis of the patients with CSVDs indicated that only age was a risk factor for the recurrence of BPPV, after controlling the other factors. We considered that CSVDs may are likely more prevalent among the elderly and is not be an independent factor in the recurrence of BPPV. Some studies have indicated the recurrence of BPPV is related to gender, CRP times, previous BPPV, and is involved side and duration of disease 7, 14, 33. However, some studies have the opposite view 12, 27, 31. In this study, we did not find a correlation between these above factors and recurrence of BPPV. Only the previous history of BPPV was close to statistical significance in the difference in recurrence rate. Increasing age as a risk factor for BPPV recurrence has been consistently identified in many studies on the topic 3, 34, 35. This may be due to change of otoconial morphology with age, which causes increases in detachment 36.

Several studies have identified that non-rotational dizziness and walking instability continues to exist after a successful repositioning of BPPV, with an incidence rate of 31.1% to 61%, and is termed as residual dizziness (RD) 37-40. Generally, a positional nystagmus test is negative, without nausea and vomiting 16, 41, 42. The duration of RD symptoms ranges from a few days to weeks, and even several months have been previously reported 12, 16, 37-39, 42. The mechanism may be due to an additional vestibular dysfunction that coexists with BPPV and causes less-specific dizziness or incomplete central adaptation, which needs a longer time after repositioning 43-45. Some researchers have even suggested that the remaining otoconial debris may not be completely repositioned, which can cause mild positional vertigo, but not enough to provoke any overt nystagmus 43, 46. Some studies have also shown that BPPV is not only a disorder of semicircular canals, but also an otolith dysfunction that may account for transient mild dizziness 47, 48. Residual vertigo has a great impact on quality of life, is a risk factor for falls and causes anxiety 42, 49.

In this study, we determined that the incidence of RD in the CSVDs group is higher compared to the non-CSVDs group, after matching on the propensity score; the differences are statistically significant. This suggests that BPPV patients with CSVDs are more likely to have RD. Chen *et al.* found a higher percentage of RD in BPPV patients with central neurologic disorders, a result which needs to be verified in a larger case-control study 50. In addition, some studies have found that CSVD is closely related to vertigo 22, 51-53. This study found that occurrence of RD is closely related to the severity of WMH. Logistic multiple regression analysis of 176 BPPV patients with CSVDs shows that severity of WMH and age are risk factors for RD. This is further supported by a study of Adamec *et al.*, who found that the presence of WMH is a predictor for chronic RD among patients with previous vestibular neuritis 54. In addition, Ahmad *et al*. found that the severity of WHM is higher among patients with unexplained dizziness compared to those with specific causes (BPPV, vestibular neuritis, vestibular migraine, Meniere's disease, and other diseases) 52. All of these results suggest that WMH likely contributes to the development of RD. The reason for this analysis may be that WMH might affect vestibular cortex and related neural networks 21. Some scholars have also suggested that WMH patients may have a cortical-subcortical disconnection syndrome 10, 52. We speculate that a damage of the neural pathway may affect central vestibular adaptation and increase risk of RD.

In addition, some studies have shown that RD may be related to the duration of vertigo prior to CRP 37, 38, 45, but there are still some controversies 39, 42. This study did not find any significant correlation between duration of BPPV and RD. The reason for the analysis may be selection bias, and that the analyzed population is patients with CSVDs. However, the recognition that age is a risk factor for RD is relatively consistent across studies 16, 35, 41, 42. Our findings indicated that there is a significant correlation between age and occurrence of RD. The vestibular function of elderly patients suffers from age-related decline 36, 42. In addition, elderly patients are more likely to develop anxiety, which has also been shown to promote occurrence of RD 5, 42. Cha *et al.* found that brain atrophy was related to long-lasting dizziness after BPPV treatment and speculated that there may be a relationship between brain atrophy and otoneurologic diseases 12. This study indicates that BPPV patients with CSVDs who develop RD have a higher incidence of brain atrophy, but the logic multivariate analysis does not find that brain atrophy is a risk factor for RD.

Therefore, it is of great clinical significance that we identify BPPV patients who are prone to RD and recurrence, as well as to intervene with treatment. In addition to CRPs, vestibular rehabilitation exercises have been shown to be effective at controlling RD symptoms and preventing BPPV recurrence 55, 56. In addition, for BPPV patients with CSVDs, whether active treatment of CSVDs is helpful to the prognosis of BPPV, requires further research to prove it. This study had some limitations. First, a larger number of patients and longer follow-up time may be needed to provide more precise values in the future. Second, we excluded patients who did not complete brain MRI, which may cause selection bias. In addition, due to outpatient medical records, the patients' information was not fully complete for some potential risk factors, including psychological factors and otoneurologic examinations. Finally, we did not quantitatively evaluate the degree of brain atrophy, CMB and EPVS, but simply classified it as presence or absence.

## Conclusion

We investigated the type and proportion of CSVD in BPPV patients with CSVDs and focused on determining the prognosis of BPPV patients with CSVDs, including recurrence rate and RD after successful CRP. WMH and LI were found to be the most prevalent types of CSVD in among BPPV patients. After controlling for other confounders using a propensity score matching approach, we found that patients with CSVDs were more likely to have RD. In addition, logistic regression analysis also indicated that increased age and WMH level were independent risk factors for RD. Accordingly, we conclude that older BPPV patients with WMHs are more likely to develop RD, and therefore need to be paid more attention. Perhaps, for BPPV patients with CSVDs, we should actively treat CSVDs at the same time as CRP treatment in order to reduce the risk of RD. We will expand the sample size in future studies and design controlled trials to further validate our findings.

## Figures and Tables

**Table 1:** Baseline clinical characteristics and the incidence of recurrence and RD in BPPV patients with and without CVSDs after matching on the propensity score

	CSVDs group n=78 (%)	Non-CSVDs group n=78 (%)	P value
Age (years)	68.15±9.23	68.73±10.15	0.598
Gender (male)	31(39.7%)	30(38.5%)	0.870
Laterality (right)	41(52.6%)	43(55.1%)	0.748
Previous BPPV (yes)	33(42.3%)	30(38.5%)	0.624
Duration of BPPV (days)	5.91±3.63	5.89±3.51	0.207
Number of CRPs	1.36±0.44	1.35±0.46	0.434
**Canal involved**			
PSC-BPPV	55(70.5%)	58(74.4%)	0.771
HSC-BPPV	21(26.9%)	19(24.4%)	
AC-BPPV	2(2.6%)	1(1.3%)	
1- year recurrence	27(34.6%)	17(21.8%)	0.075
RD	36(46.2%)	21(26.9%)	0.013

Abbreviations: anterior semicircular (ASC); benign paroxysmal positional vertigo (BPPV); canalith repositioning procedure/maneuvers (CRP); cerebral small vessel disease (CVSD); horizontal semicircular (HSC); posterior semicircular (PSC); residual dizziness (RD).

**Table 2:** Analysis of recurrence rate and RD incidence in 176 BPPV patients with CSVDs

Variables	Recurrence			RD		
	Yes	No	P value	Yes	No	P value
Subjects	61(34.7%)	115(65.3%)		100(56.8%)	76(43.2%)	
Age^ b^	74(7.5)	62(19)	<0.001	72(9.75)	54(18.75)	<0.001
**Gender**						
Male	22(29.7%)	52(70.3%)	0.242	39(52.7%)	35(47.3%)	0.348
Female	39(38.2%)	63(61.8%)		61(59.8%)	41(40.2%)	
**Laterality**						
Right	32(34.8%)	60(65.2%)	0.971	54(58.7%)	38(41.3%)	0.599
Left	29(34.5%)	55(65.5%)		46(54.8%)	38(45.2%)	
**Previous BPPV**						
Yes	34(41.5%)	48(58.5%)	0.076	46(56.1%)	36(43.9%)	0.857
No	27(28.7%)	67(71.3%)		54(57.4%)	40(42.6%)	
Duration of BPPV(days)^ a^	6.26±3.20	6.50±3.30	0.641	6.52±3.33	5.88±3.15	0.199
Number of CRPs^ b^	2(1)	1(1)	0.138	2(1)	1(1)	0.500
**Canal involved**			0.113			0.730
PSC-BPPV	35(57.4%)	82(71.3%)		64 (64%)	53 (69.7%)	
HSC-BPPV	24(39.3%)	28(24.3%)		32 (32%)	20 (26.3%)	
AC-BPPV	2(2.4%)	5(4.3%)		4 (4%)	3 (3.9%)	
**CSVDs**						
WMH^ b^	5(3)	3(6)	0.030	5(3)	0(4)	<0.001
LI^ b^	4(3)	2(5)	0.025	3(5)	2(5)	0.119
**Brain atrophy (Yes)**	22(40.0%)	33(60.0%)	0.315	41(74.5%)	14(25.5%)	0.001
(No)	39(32.2%)	82(67.8%)		59(48.8%)	62(51.2%)	
**CMB (Yes)**	7(43.8%)	9(56.3%)	0.423	12(75.0%)	4(25.0%)	0.124
(No)	54(33.8%)	106(66.3%)		88(55.0%)	72(45.0%)	
**EPVS (Yes)**	8(38.1%)	13(61.9%)	0.724	14(66.7%)	7(33.3%)	0.332
(No)	53(34.2%)	102(65.8%)		86(55.5%)	69(44.5%)	

Abbreviations: anterior semicircular (ASC); benign paroxysmal positional vertigo (BPPV); cerebral microbleed (CMB); canalith repositioning procedure/maneuvers (CRP); cerebral small vessel disease (CVSD); enlarged perivascular space (EPVS); horizontal semicircular (HSC); lacunar infarction (LI); posterior semicircular (PSC); residual dizziness (RD); white matter hyperintensities (WMH); ^a^ Mean±standard deviation (SD), ^b^ Median (quartile range, QR).

## Abbreviations

ASC: anterior semicircular
BPPV: benign paroxysmal positional vertigo
CMB: cerebral microbleed
CRP: canalith repositioning procedure/maneuvers
CVSD: cerebral small vessel disease
EPVS: enlarged perivascular space
HSC: horizontal semicircular
LI: lacunar infarction
PSC: posterior semicircular
PSM: propensity score matching
RD: residual dizziness
WMH: white matter hyperintensities
